# Global A-to-I RNA editing during myogenic differentiation of goat MuSCs

**DOI:** 10.3389/fvets.2024.1439029

**Published:** 2024-10-09

**Authors:** Xiaoli Xu, Mancheng Zhang, Siyuan Zhan, Yuan Chen, Chengqi Wei, Jiaxue Cao, Jiazhong Guo, Dinghui Dai, Linjie Wang, Tao Zhong, Hongping Zhang, Li Li

**Affiliations:** Farm Animal Genetic Resources Exploration Innovation Key Laboratory of Sichuan Province, College of Animal Science and Technology, Sichuan Agricultural University, Chengdu, China

**Keywords:** A-to-I editing, high-throughput sequencing, skeletal muscle satellite cells (MuSCs), myogenic differentiation, goats

## Abstract

**Background:**

RNA editing, especially A-to-I editing sites, is a common RNA modification critical for stem cell differentiation, muscle development, and disease occurrence. Unveiling comprehensive RNA A-to-I editing events associated with myogenesis of the skeletal muscle satellite cells (MuSCs) is essential for extending our knowledge of the mechanism underpinning muscle development.

**Results:**

A total of 9,632 RNA editing sites (RESs) were screened in the myoblasts (GM), myocytes (DM1), and myotubes (DM5) samples. Among these sites, 4,559 A-to-I edits were classified and further analyzed. There were 3,266 A-to-I sites in the protein-coding region, out of which 113 missense sites recoded protein. Notably, five A-to-I sites in the 3′ UTR of four genes (*TRAF6*, *NALF1*, *SLC38A1*, *ENSCHIG00000019092*) altered their targeted miRNAs. Furthermore, a total of 370 A-to-I sites with different editing levels were detected, including *FBN1*, *MYH10*, *GSK3B*, *CSNK1D*, and *PRKACB* genes. These genes were predominantly enriched in the cytoskeleton in muscle cells, the hippo signaling pathway, and the tight junction. Furthermore, we identified 14 hub genes (*TUFM*, *GSK3B*, *JAK2*, *RPSA*, *YARS1*, *CDH2*, *PRKACB*, *RUNX1*, *NOTCH2*, *CDC23*, *VCP*, *FBN1*, *RARS1*, *MEF2C*) that potentially related to muscle development. Additionally, 123 stage-specific A-to-I editing sites were identified, with 43 sites in GM, 25 in DM1, and 55 in DM5 samples. These stage-specific edited genes significantly enriched essential biological pathways, including the cell cycle, oocyte meiosis, motor proteins, and hedgehog signaling pathway.

**Conclusion:**

We systematically identified the RNA editing events in proliferating and differentiating goat MuSCs, which was crucial for expanding our understanding of the regulatory mechanisms of muscle development.

## 1 Background

RNA editing is one of the most crucial post-transcriptional mechanisms, which alters the levels and structures of transcripts through substituting nucleotides. Recently, a large number of RNA editing sites (RESs) have been identified in pigs (1), sheep (2), goats (3), bovine (4), chicken (5), and humans (6), demonstrating the importance of this modification in multiple tissues such as skeletal muscle, brain, heart, and liver. As a predominant type of RNA editing, adenosine-to-inosine (A-to-I), regulated by adenosine deaminases acting on RNA (ADARs) (7), deaminates A to I in double-stranded RNAs (dsRNAs) and consequently is recognized as guanosine (G) by the cell machinery during translation (8, 9). RNA A-to-I editing almost occurs in the non-protein-coding regions (5′ UTR, 3′ UTR, and intron) of ADAR targets, whereas the protein-coding region is relatively rare (10, 11). A-to-I is crucial for vertebrate development, which controls gene expression by non-synonymous substitutions, influencing alternative splicing, microRNA target recognition, and other biological processes (12–16).

Skeletal muscle is the most abundant tissue in mammals that is governed by myogenesis during embryonic and postnatal growth (17, 18), which is a highly orchestrated cellular interaction including myoblasts that exit the cell cycle and differentiate into myocytes and myotubes (19). Skeletal muscle satellite cells (MuSCs), a population of stem cells located beneath the basement membrane of myofiber, are activated by injury or stress to repair muscle tissue via gradual proliferation, differentiation, fusion, and maturation (20). These processes are controlled by myogenic genes (myogenic regulatory factors, paired box) (21, 22), non-coding RNAs (microRNAs, long non-coding RNAs, circular RNAs) (23–25), and epigenetic modifications (26, 27).

Many RNA editing sites have been identified in stem cell differentiation (28) and skeletal muscle development (3). As a common modification in RNA, A-to-I editing represents a new type of post-transcriptional gene regulation, which is tissue-specific and spatiotemporally specific (29, 30). However, how RNA editing is characterized remains unclear, especially in the A-to-I sites involved in myogenic differentiation. In this study, we identified RNA editing sites that occur during the critical transition of goat MuSCs from myoblast (GM) to myocyte (DM1) and myotube (DM5) stages. Our findings unveiled the A-to-I editing events in myogenic differentiation and extended our knowledge of skeletal muscle development.

## 2 Materials and methods

### 2.1 Datasets and samples

The MuSCs and blood samples were collected from the Chengdu Brown goat from the Dayi farm (Sichuan, China), and RNA-seq (PRJNA779184) and DNA-seq (PRJNA548681) were performed for them. The MuSCs were harvested from the longissimus dorsi muscle, subsequently cultured in proliferation medium (GM, myoblasts, *n* = 3) until they were 80% confluent, and maintained for 1 day (DM1, myocytes, *n* = 3) and 5 days (DM5, myotubes, *n* = 3) under differentiation conditions. Our previous publication comprehensively explained the procedures involved in sample collection and strand-specific RNA-seq data (31).

### 2.2 Reads alignment and variant calling

#### 2.2.1 Trimming

We trimmed adaptors and low-quality reads using fastp v0.23.2 with the parameters “-w 20 -q 20 -u 50 -n 15 -l 45.”

#### 2.2.2 Alignment

We aligned the high-quality reads obtained from DNA-seq to the Ensembl goat reference genome ARS1 (release-105) using the BWA-MEM method with BWA v0.7.17.^1^ The RNA-seq data was also aligned to the goat reference genome using HISAT2 v2.1.0. The software’s built-in Python scripts, extract_splice_sites.py, and extract_exons.py, extracted the splice sites and exon coordinates from the goat genome annotation file (GTF, release-105). Subsequently, the hisat2-build command with the options --ss and --exon was used to create the genome index. Finally, the high-quality reads were aligned to the goat reference genome.

#### 2.2.3 BAM process

The SAM files generated from aligning the RNA-seq and DNA-seq data to the reference genome were converted to a BAM format and sorted using samtools v1.16.1. Then, the MarkDuplicates script from the Picard v1.141 program was used to mark the duplicate reads that map to the same position.

#### 2.2.4 Variant calling

To minimize random systematic errors caused by sequencing instruments, we retrieved goat single-nucleotide polymorphisms (SNPs) from published databases, including goat reference genome and variant call format (VCF), as well as our own DNA-seq data of this goat breed. Subsequently, we recalibrated base quality scores for each alignment file using BaseCalibrator and ApplyBQSR from GATK v4.1.8.0 and merged them. Then, we called variants using HaplotypeCaller in GATK and separated SNPs from short insertion and deletion (INDEL) fragments using SelectVariants. This step was not subjected to any filtering to obtain a comprehensive genomic variation result, enabling the most stringent control for subsequent identification of variations.

### 2.3 Identifying RNA editing sites

#### 2.3.1 Detection of RESs

The REDItoolDnaRna.py script from REDItools v0.19.1 was used to detect raw RNA editing sites. The script was executed with the following parameters: -c 10,1 -q 25,25 -m 20,20 -s 2 -g 1 -u -a 6-0 -v 2 -n 0.0 -N 0.0 -V.

#### 2.3.2 Filtration of RESs

The selectPositions.py script provided by REDItools v0.19.1 was used for preliminary filtering. The script was executed with the following parameters: -c 10 -C 5 -v 2 -V 0 -f 0.01 -F 0.95 -e -u. Then, stringent filtering was applied to the obtained RNA editing sites. The intersected functionality of BEDtools v2.30.0 (32) was used to filter out the editing sites that overlap with the goat SNP database (release-105) and VCF. Additionally, RNA editing sites supported by less than three samples are excluded.

#### 2.3.3 Annotation of RESs

Utilizing the SnpEff v5.1, the precise localization of RNA editing sites was performed based on the GTF and the goat reference genome. The software accurately determined the positions of RNA editing sites and categorized them into specific regions, including 3′ UTR, 5′ UTR, intergenic regions, introns, and exons.

### 2.4 Sequence preference of RESs

The BEDtools v2.30.0 (32) was used to obtain sequences of 21 bp in total, consisting of 10 bp upstream and 10 bp downstream regions, from the RNA editing sites in the goat genome. The obtained sequences were then analyzed using the R package ggseqlogo to calculate the frequency of each nucleotide, which was used to investigate the base preferences of RNA editing within various gene features.

### 2.5 Stage differential and specific comparisons of RESs

We performed variance analysis in R to compare the differences in RNA editing levels among three stages, GM, DM1, and DM5, with a significance threshold (*p* < 0.05). RNA editing sites that were identified as edited only in a specific stage and not detected in the other stages were referred to as stage-specific editing sites. The correlation analysis between the number of RESs and chromosome length, RNA editing levels and chromosome length, and RNA editing levels and gene expression was performed using the Spearman method from the R package ggpubr. The correlation plots were generated using the ggplot2.

### 2.6 The prediction of target miRNAs of genes

To investigate the miRNA-targeted editing changes in the 3′ UTR of the genes, miRanda v3.3 was used to predict miRNA targets on the unedited and edited 3′ UTR sequences. Goat miRNA data were downloaded from miRbase (http://www.mirbase.org/, accessed on April 10, 2023). The RNA editing sites were used to obtain 10 bp sequences on each side (a total of 21 bp) using the getfasta tool in BEDtools. The miRanda v3.3 software was employed with the parameters “-sc 140 -en -10” for the prediction of binding sites. To detect changes in target miRNA before and after editing, the nucleotides at the editing sites in the gene were replaced with the edited type. Predicting miRNA target genes using the same method to infer miRNA functions. Gene 3′ UTR position information was obtained from the goat GTF, and the 3′ UTR sequence of the gene was extracted using the getfasta tool of BEDtools.

### 2.7 Functional enrichment analysis of RESs-related genes

To investigate the functionality of candidate RESs, based on the human database (org.Hs.eg.db v3.17.0), we conducted gene ontology (GO) enrichment and Kyoto Encyclopedia of Genes and Genomes (KEGG) pathway analysis using Fisher’s test.

### 2.8 Protein interaction network of RES-related genes

The human database from the STRING (https://string-db.org, accessed on May 12, 2023) was used to construct a protein–protein interaction network for the genes with differential sites and visualized by Cytoscape v3.10.0.

## 3 Results

### 3.1 Identifying RNA editing sites in goat MuSCs myogenesis

**Figure 1:**
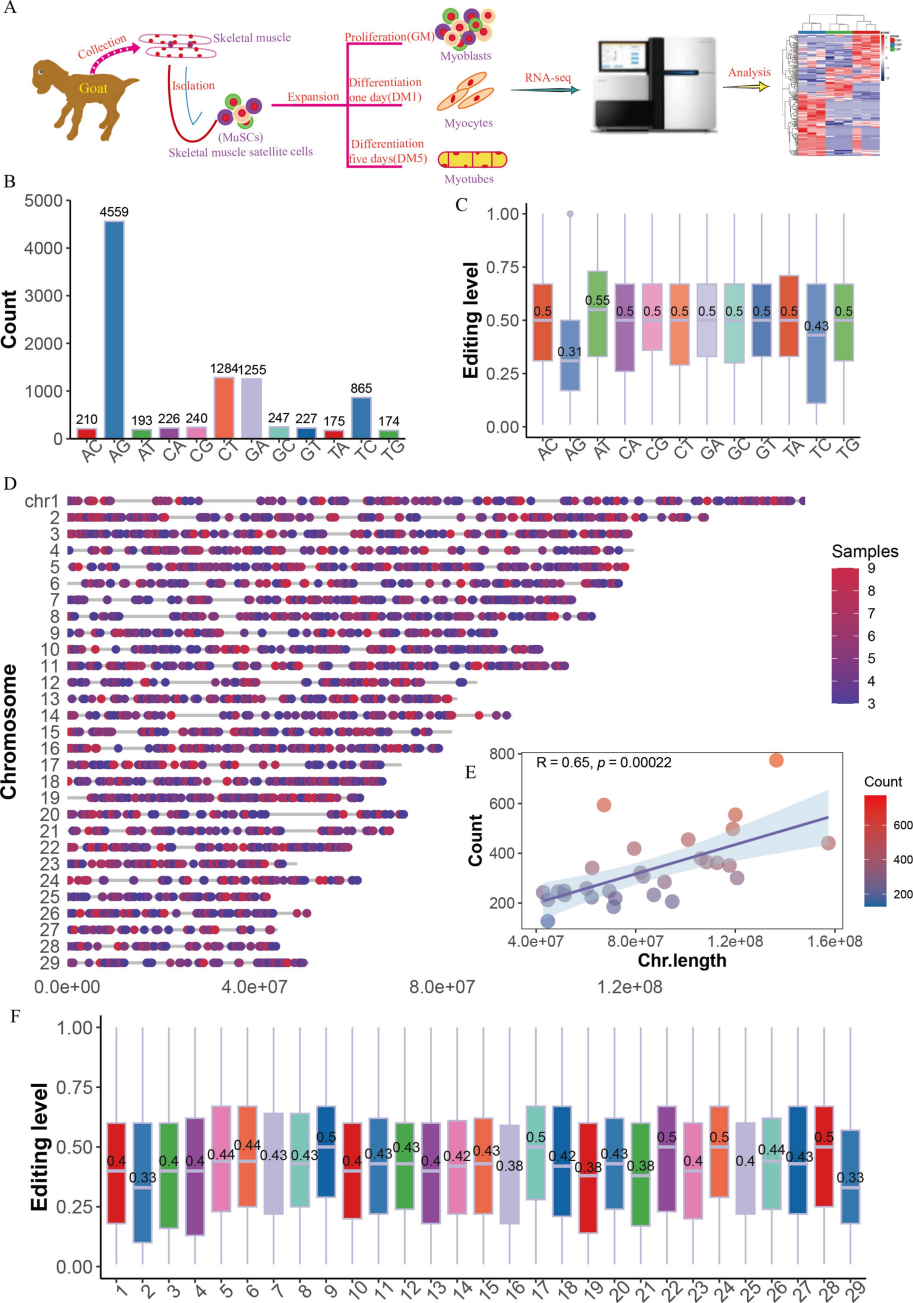
The distribution of RNA editing sites in goat MuSCs. (A) Overall experimental design. The numbers (B) and levels (C) of 12 RNA editing types. (D) The genome distribution of RNA editing sites (RESs). (E) The relationship between chromosome length and the number of RESs. (F) The editing levels across chromosomes.

In this study, we used nine strand-specific RNA-seq data of goat MuSC differentiation samples for three stages: myoblasts that had high proliferation with mononuclear (GM), myocytes that differentiated with mononuclear (DM1), and myotubes that fusion into multinuclear (DM5) (Figure 1A). We filtered out the 105,271 SNPs and the sites supported by less than three samples, and screened a total of 9,632 RNA editing sites (RESs, Supplementary Table S1), which were categorized into 12 types, with the most dominant editing numbers of A-to-G (AG, 4,559 sites) (Figure 1B). Intriguingly, the editing levels of AG (0.31) were the lowest, compared with other editing types with levels approximately 0.5 (Figure 1C). Additionally, RESs were mainly present on the reverse strand (Supplementary Figure S1B) and unevenly distributed on each chromosome (Figure 1D). The number of RESs was positively correlated with chromosome length (*R* = 0.65 *p* = 0.00022, Figure 1E), with the fewest sites on chromosome 27 (127 sites) and the highest number on chromosome 2 (774 sites) (Supplementary Figure S1A), but insignificant associated with editing levels (*R* = −0.19, *p* = 0.31, Figure 1F; Supplementary Figure S1C).

### 3.2 Charactering A-to-I editing sites in the myogenesis of MuSCs

**Figure 2:**
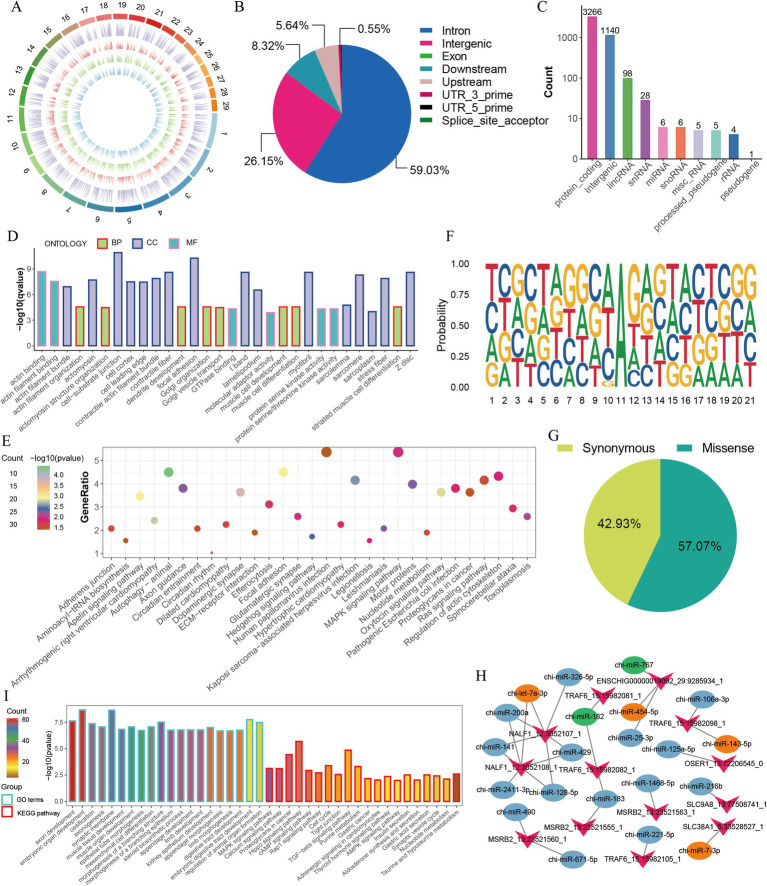
Characterization of A-to-I editing sites in MuSCs. (A) The chromosome distribution of detected A-to-I editing sites in each MuSC differentiation stage. The circuits represent total editing sites in all stages and editing sites identified in GM, DM1, and DM5 samples from the outer to the inner. (B) The distribution of genomic location of A-to-I editing sites. (C) The distribution of gene types of A-to-I editing sites. (D) GO enrichment of the protein-coding genes. (E) KEGG enrichment of the protein-coding genes. (F) Sequence preference for A-to-I editing sites (±10 bp). (G) The proportion of amino acid mutation types. (H) The changes in the number of miRNAs with RNA editing in the 3′ UTR of genes. Green represents disappeared miRNAs after editing, and orange represents new miRNAs after editing. (I) GO and KEGG enrichment of the target genes of six disappeared and emerged miRNAs.

We further analyzed the characteristics and functions of the 4,559 A-to-I (AG) editing sites (Supplementary Table S2), which were unevenly distributed on the chromosomes in the GM, DM1, and DM5 samples (Figure 2A). Most A-to-I editing sites were present in three or four samples (Supplementary Figure S1D), and many sites exhibited editing levels below 0.5 (Supplementary Figure S1E). A-to-I editing sites were primarily located in the intron region (59.03% of the sites), followed by intergenic areas (26.15%) and downstream (8.32%) (Figure 2B). Additionally, the A-to-I editing sites were annotated for 3,266 protein-coding genes, along with non-coding RNAs such as lincRNAs (98 genes), snoRNAs (six genes), snRNAs (28 genes), and miRNAs (six genes) (Figure 2C). Moreover, we found that most of the genes (85.13%) embedded one to three editing sites, of which 56.96% of genes had one site, 19.75% had two sites, and 5.06% harbored three sites (Supplementary Figure S1F). We subsequently performed GO and KEGG enrichment analyses for the 3,266 protein-coding genes, including *ADAMTS5*, *HDAC4*, *ADAM12*, *FGFR2*, *MEF2C*, *MEF2A*, and *MYH11*. They were enriched in GO terms, primarily encompassed processes related to muscle cell differentiation and muscle cell development (Figure 2D and Supplementary Table S3). The KEGG pathways highlighted the involvement of the Ras signaling pathway, MAPK signaling pathway, and hedgehog signaling pathway, which are crucial for skeletal muscle development (Figure 2E and Supplementary Table S4).

Previous studies suggested the sequence preference between A-to-I editing and ADAR binding (33). Accordingly, we observed a distinctive sequence pattern in the 10-base pair upstream and downstream regions of A-to-I editing sites. This pattern revealed a depletion and enrichment of G bases on the upstream and downstream regions near the A-to-I editing sites (Figure 2F). Similar to DNA mutations, A-to-I editing can modify the amino acid sequence, influence splicing variations, and regulate RNA expression (12, 13). Among the 198 sites in the coding region, 42.93% (85) of them led to synonymous mutations, and 57.07% (113) resulted in missense mutations (Figure 2G). Of 113 editing sites that altered the amino acid sequence, one site caused variable splicing variations (Supplementary Table S5), potentially influencing protein function. Moreover, 13 sites altered the 3′ UTR of the genes (Supplementary Table S6).

Since A-to-I editing sites potentially affect miRNA-mRNA interactions (14, 34, 35), we identified five editing-related changes in genes such as *SLC38A1*, *TRAF6*, *NALF1*, and *ENSCHIG00000019092*, with miRNAs targeted in their 3′ UTR (Supplementary Table S6). Once these four genes were edited, the potential targeting of chi-miR-182 and chi-miR-767 on 3′ UTR of their mRNA disappeared, and four new miRNAs (chi-miR-454-5p, chi-miR-7-3p, chi-let-7a-3p, chi-miR-143-5p) appeared instead (Figure 2H). For example, *TRAF6*, associated with muscle atrophy (36, 37), led to a disappearance in target chi-miR-182 but an appearance in chi-miR-143-5p after editing. We also predicted the target genes and their functions of five miRNAs that changed due to AG editing (Figures 2H,I). GO and KEGG terms of those target genes were enriched in embryonic organ development, muscle tissue development, MAPK signaling pathway, hippo signaling pathway, and TGFβ signaling pathway (Figure 2I). These findings suggest that A-to-I editing may participate in the proliferation and differentiation of MuSCs by modifying the amino acid sequence and regulating miRNA-mRNA interactions.

### 3.3 Specific and differential A-to-I editing sites in MuSC myogenesis

**Figure 3:**
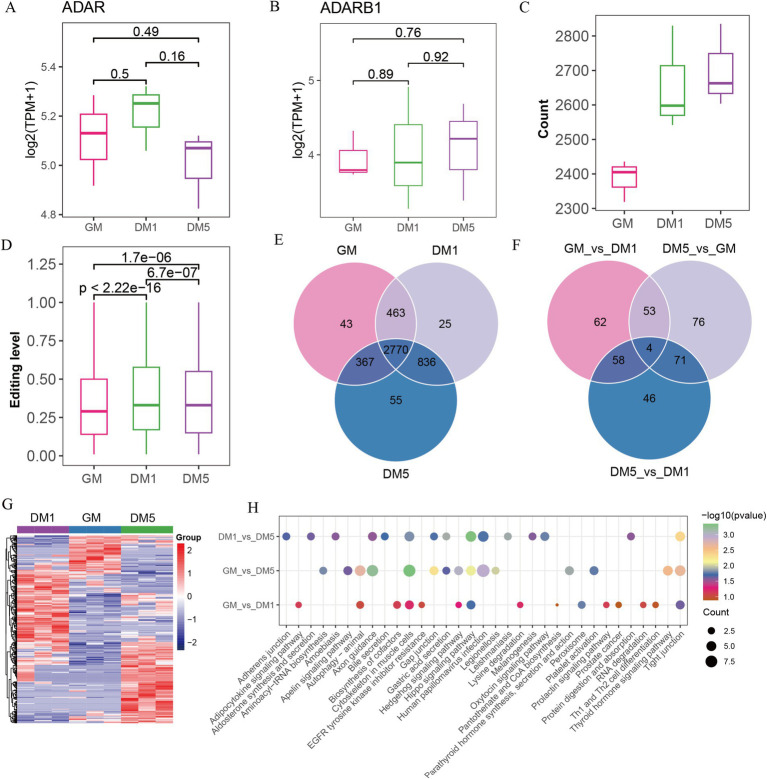
Identifying A-to-I editing sites in GM, DM1, and DM5 samples. (A) The expression of (A) *ADAR* and (B) *ADARB1*. (C) The numbers of A-to-I editing sites in GM, DM1, and DM5 stages. (D) The levels of A-to-I editing sites in GM, DM1, and DM5 stages. (E) The specific and overlap of A-to-I editing sites among three stages during MuSCs differentiation. (F) Venn diagram of the differential A-to-I editing sites among three stages. (G) Heatmap of the differential A-to-I editing sites. (H) KEGG pathways of genes with differential editing sites in each stage.

A-to-I editing is mediated by the ADAR family members (ADAR, ADARB1, and ADARB2) and human ADAD (adenosine deaminase domain-containing) family members (TENR, TENRL), possessing common functional domains (8). We found that expressions of ADAD family members were absent (data not shown), but two ADAR family members, for example, ADAR and ADARB1, were expressed. *ADAR* was initially increased and subsequently decreased from GM to DM1 and DM5 samples, while *ADARB1* increased from DM1 to DM5 samples (Figures 3A,B), coinciding with the observed number changes of A-to-I editing sites (Figure 3C) and the editing levels from GM to DM1 samples (Figure 3D).

Among the 4,559 A-to-I editing sites, 2,770 overlapped in the GM, DM1, and DM5 samples (Figure 3E). The genes in these sites were enriched in various GO terms, including cell leading edge, actin binding, and cell-substrate junction. In addition, these genes were associated with KEGG pathways such as the WNT signaling pathway, motor proteins, and cytoskeleton in muscle cells (Supplementary Figure S2A).

Based on the differential analysis, we found a total of 370 differed A-to-I editing sites occurring in genes (*FBN1*, *MYH10*, *GSK3B*, *CSNK1D*, *PRKACB*, etc.), with 177 sites in GM_vs_DM1, 204 sites in GM_vs_DM5, and 179 sites in DM1_vs_DM5 (Supplementary Table S7 and Supplementary Figure S1G). The GM_vs_DM1, GM_vs_DM5, and DM1_vs_DM5 shared four differential sites in genes *NXN*, *TBC1D2*, *ENSCHIG00000010550*, and *ENSCHIG00000002773* (Figure 3F). These differential editing sites consistently replicated the three stages (Figure 3G). Moreover, the GM_vs_DM5 differential sites in genes such as *ADAM12*, *MEF2C*, and *ADAMTS5* were enriched in GO terms associated with the NADH metabolic process and striated muscle cell differentiation (Supplementary Table S8). KEGG pathways revealed that the GM_vs_DM1 differential sites were enriched in the biosynthesis of cofactors, while DM1_vs_DM5 differential sites in the protein digestion and absorption and oxytocin signaling pathway. The GM_vs_DM5 differential sites were involved in aminoacyl-tRNA biosynthesis. All of them were enriched in the cytoskeleton in muscle cells, the hippo signaling pathway, and the tight junction (Figure 3H).

Additionally, we identified 123 stage-specific sites, including 43 GM-specific A-to-I sites in genes (e.g., *GSK3B*, *EPHB1*, *AFAP1*, *ADAM12*, etc.), 25 DM1-specific editing sites in genes (e.g., *ADAMTS5*, *MOV10*, *CDC23*, *SDC2*, *DHRS3*, etc.), and 55 DM5-specific editing sites in genes, including *TAGLN3*, *SREBF2*, *ART3*, *MEF2C*, etc. (Figure 3E and Supplementary Table S9). Genes harboring these specific editing sites displayed enrichment in various GO entries. Specifically, GM-specific sites were involved in the cellular amino acid metabolic process and protein autophosphorylation, while DM1-specific sites were associated with p-body and collagen-containing extracellular matrix (Supplementary Table S8). Furthermore, the KEGG pathways revealed that GM-specific sites were prominent in the hedgehog signaling pathway, cysteine and methionine metabolism, and arginine and proline metabolism. The cell cycle and oocyte meiosis have been highlighted in DM1-specific sites, whereas motor proteins and folate biosynthesis were abundant in DM5-specific sites (Supplementary Figure S2B).

### 3.4 Protein interaction network of genes containing A-to-I editing sites

**Figure 4:**
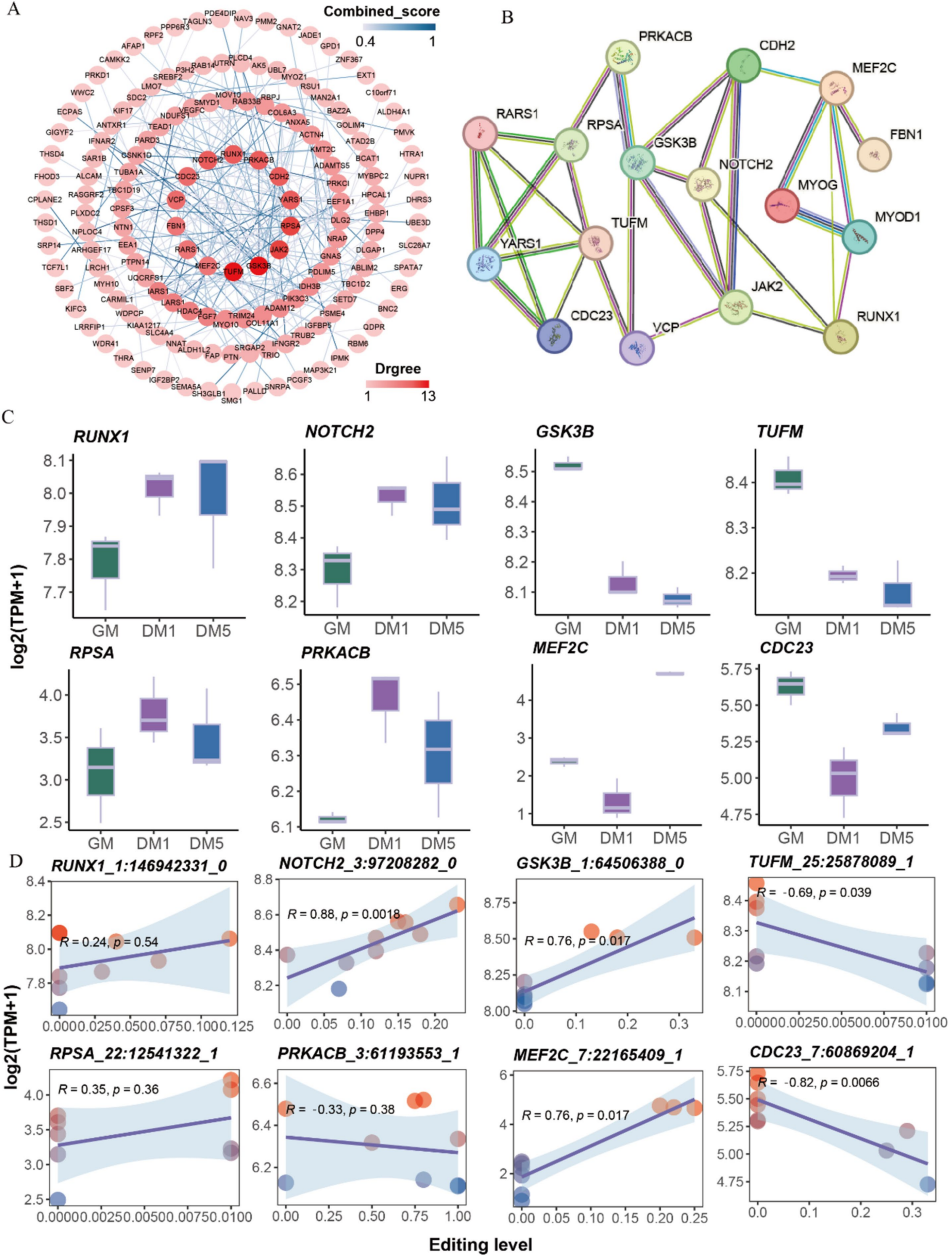
Protein interaction network analysis. (A) The protein interaction network of genes with differentially A-to-I edited. (B) The protein interaction network of 14 hub genes and two myogenic differentiation marker genes (*MYOD1*, *MYOG*). (C) The expressions of eight hub genes with differential A-to-I editing sites in each sample. (D) The interaction relationship between the expressions of eight hub genes and editing levels of differential A-to-I editing sites.

The interaction relationship between genes with differential sites was performed using Cytoscape v3.10.0. A total of 14 hub genes, including *TUFM*, *GSK3B*, *JAK2*, *RPSA*, *YARS1*, *CDH2*, *PRKACB*, *RUNX1*, *NOTCH2*, *CDC23*, *VCP*, *FBN1*, *RARS1*, and *MEF2C* were detected (Figure 4A). Additionally, we examined the interaction between these hub genes and marker genes (*MYOD1*, *MYOG*) associated with muscle differentiation. The results revealed that *MYOD1* and *MYOG*, two well-known regulators of skeletal muscle development, strongly interacted directly with *MEF2C* and *RUNX1* and indirectly interacted with other hub genes (Figure 4B).

The expressions of hub genes such as *RUNX1* and *NOTCH2* were elevated along with the myogenic differentiation of MuSCs, while *GSK3B*, *TUFM*, and *JAK2* were decreased. The expressions of *RPSA*, *PRKACB*, and *FBN1* peaked at the DM1 stage. Conversely, the expressions of *MEF2C*, *CDC23*, *CDH2*, *YARS1*, *RARS1*, and *VCP* were the lowest at the DM1 stage (Figure 4C; Supplementary Figure S3A). Interestingly, A-to-I editing significantly increased *NOTCH2*, *GSK3B*, and *MEF2C* expression, significantly decreasing *TUFM* and *CDC23* expression (Figure 4D; Supplementary Figure S3B). These findings suggest that A-to-I editing plays a role in regulating the expressions of these hub genes involved in muscle development.

## 4 Discussion

RNA editing events are known to be crucial for the development of various organisms, including humans (38), yak (39), goat (3), sheep (2), and chicken (5). For instance, the number of A-to-I editing sites in goat skeletal muscle has been reported to decrease after birth (3). MuSCs play vital roles in muscle recovery and regeneration (40). However, investigating RNA editing sites during MuSC differentiation has been limited. In this study, we analyzed the A-to-I editing sites during the MuSC proliferation (GM) and differentiation (DM1/DM5) process, which was crucial for expanding the regulatory mechanisms of muscle development.

We identified 9,632 editing sites that were unevenly distributed across the chromosomes. Among these, 4,559 sites were A-to-I editing sites, preferentially located on the intron and rarely in coding regions (28, 41, 42). Additionally, AG editing type also occupied a significant portion, which was consistent with the trend shown in previous studies in goats (3, 43), chicken (44), humans (45), and sheep (2). We also found 113 missense and 85 nonsense A-to-I editing sites, with a significant proportion having the potential to recode proteins. This indicates that these editing sites may play crucial roles in differentiating MuSCs. While a large number of RNA editing sites have been identified in humans (3,041,422 sites) (30), bovine (1,600 sites) (4), and pig (198,892 sites) (29), only a few of them have been extensively studied. For example, ADAR1-mediated A-to-I RNA editing occurs at the U1 snRNA binding site of the 5′ splice site, preventing Alu exonization and promoting mature mRNA production during skeletal muscle development (46). Furthermore, the effect of RNA editing on miRNA-mRNA relationships has been revealed, with only five editing sites in mRNA 3′ UTR altering miRNA recognition, resulting in the repression of protein translation and mRNA expression (47, 48). Once editing appeared in mRNA 3′ UTR of genes, their targeted miRNAs were potentially altered. For example, miR-182 decreases during muscle atrophy and suppresses atrophy by targeting *FoxO3* in C2C12 cells (49); its targeted sites disappeared after editing. Meanwhile, sites for miR-7 emerged by editing; miR-7 itself decreases myoblast proliferation and differentiation by inhibiting *TCF12* and *KLF4* genes in C2C12 and chicken primary myoblasts, respectively (50, 51).

Skeletal myogenesis is a complex process involving cell proliferation, differentiation, and fusion and is regulated by an intricate gene regulatory network. We identified 370 differential A-to-I editing sites in genes such as *ADAM12*, *MEF2C*, and *ADAMTS5* that enriched the NADH metabolic process and striated muscle cell differentiation, closely related to muscle metabolism (52) and development (53). Moreover, we detected 14 hub genes (*TUFM*, *GSK3B*, *JAK2*, *RPSA*, *YARS1*, *CDH2*, *PRKACB*, *RUNX1*, *NOTCH2*, *CDC23*, *VCP*, *FBN1*, *RARS1*, *MEF2C*) that are involved in regulating cell cycle (54, 55), cell differentiation (56, 57), skeletal muscle regeneration (58, 59), and mitochondrial metabolism (60) across the three stages of MuSC differentiation. Consistent with previous studies indicating the critical role of A-to-I RNA editing during early human embryogenesis in a stage-specific manner (38, 61), we identified 123 stage-specific A-to-I editing sites in the GM, DM1, and DM5 stages. These editing sites are involved in cell cycle regulation, motor proteins, and the hedgehog signaling pathways, all of which play essential roles in muscle development (62, 63). For instance, *ADAM12* (meltrin-α), a muscle regeneration marker, was detected in the GM stage and was primarily expressed in muscle and bone during mouse embryogenesis (64). Mice deficient in *ADAM12* had a mortality rate of 30% within the first few weeks after birth and its re-expression was observed during the fusion of myogenic cells with newly formed fibers (65, 66). Similarly, *ADAMTS5*, a gene specific to the DM1 stage, impaired C2C12 myoblast fusion when its expression was knocked down (67). *MEF2C*, a gene specific to the DM5 stage, played a positive regulatory role in muscle differentiation and regeneration (57, 68). However, further studies are warranted to elucidate the specific roles of these genes in MuSCs and their underlying regulatory mechanisms.

## 5 Conclusion

This study systematically identified the RNA editing events in proliferating and differentiating goat MuSCs. A-to-I editing potentially modified amino acid sequences and miRNA target binding. We revealed 123 specific A-to-I editing sites and 370 differential A-to-I editing sites. Additionally, 14 hub genes are essential in regulating cell cycle, cell differentiation, and skeletal muscle development. These findings strongly suggest that these identified A-to-I editing sites play a crucial role in skeletal muscle development, although their specific functions remain fully elucidated.

## Data Availability

The raw sequencing data are available. These data can be found at: https://www.ncbi.nlm.nih.gov/, PRJNA779184, and PRJNA548681.

## Footnotes

^1^https://github.com/lh3/bwa

## References

[1] Martínez-Montes AM Fernández A Pérez-Montarelo D Alves E Benítez RM Nuñez Y. Using RNA-seq SNP data to reveal potential causal mutations related to pig production traits and RNA editing. Anim Genet. (2017) 48:151–65. doi: 10.1111/age.12507 27642173

[2] Zhang Y Han D Dong X Wang J Chen J Yao Y. Genome-wide profiling of RNA editing sites in sheep. J Anim Sci Biotechnol. (2019) 10:31. doi: 10.1186/s40104-019-0331-z, PMID: 30918658 PMC6419479

[3] Yang L Li L Kyei B Guo J Zhan S Zhao W. Systematic analyses reveal RNA editing events involved in skeletal muscle development of goat (*Capra hircus*). Funct Integr Genomics. (2020) 20:633–43. doi: 10.1007/s10142-020-00741-0, PMID: 32447468

[4] Bakhtiarizadeh MR Salehi A Rivera RM. Genome-wide identification and analysis of A-to-I RNA editing events in bovine by transcriptome sequencing. PLoS One. (2018) 13:e0193316. doi: 10.1371/journal.pone.0193316, PMID: 29470549 PMC5823453

[5] Frésard L Leroux S Roux PF Klopp C Fabre S Esquerré D. Genome-wide characterization of RNA editing in chicken embryos reveals common features among vertebrates. PLoS One. (2015) 10:e0126776. doi: 10.1371/journal.pone.0126776, PMID: 26024316 PMC4449034

[6] Tan MH Li Q Shanmugam R Piskol R Kohler J Young AN. Dynamic landscape and regulation of RNA editing in mammals. Nature. (2017) 550:249–54. doi: 10.1038/nature24041, PMID: 29022589 PMC5723435

[7] Nishikura K. Functions and regulation of RNA editing by ADAR deaminases. Annu Rev Biochem. (2010) 79:321–49. doi: 10.1146/annurev-biochem-060208-105251, PMID: 20192758 PMC2953425

[8] Nishikura K. A-to-I editing of coding and non-coding RNAs by ADARs. Nat Rev Mol Cell Biol. (2016) 17:83–96. doi: 10.1038/nrm.2015.4, PMID: 26648264 PMC4824625

[9] Walkley CR Li JB. Rewriting the transcriptome: adenosine-to-inosine RNA editing by ADARs. Genome Biol. (2017) 18:205. doi: 10.1186/s13059-017-1347-3, PMID: 29084589 PMC5663115

[10] Li JB Levanon EY Yoon JK Aach J Xie B Leproust E. Genome-wide identification of human RNA editing sites by parallel DNA capturing and sequencing. Science. (2009) 324:1210–3. doi: 10.1126/science.1170995, PMID: 19478186

[11] Stapleton M Carlson JW Celniker SE. RNA editing in *Drosophila melanogaster*: new targets and functional consequences. RNA. (2006) 12:1922–32. doi: 10.1261/rna.254306, PMID: 17018572 PMC1624909

[12] Gott JM Emeson RB. Functions and mechanisms of RNA editing. Annu Rev Genet. (2000) 34:499–531. doi: 10.1146/annurev.genet.34.1.499 11092837

[13] Laurencikiene J Källman AM Fong N Bentley DL Ohman M. RNA editing and alternative splicing: the importance of co-transcriptional coordination. EMBO Rep. (2006) 7:303–7. doi: 10.1038/sj.embor.7400621, PMID: 16440002 PMC1456888

[14] Kawahara Y Zinshteyn B Sethupathy P Iizasa H Hatzigeorgiou AG Nishikura K. Redirection of silencing targets by adenosine-to-inosine editing of miRNAs. Science. (2007) 315:1137–40. doi: 10.1126/science.1138050, PMID: 17322061 PMC2953418

[15] Borchert GM Gilmore BL Spengler RM Xing Y Lanier W Bhattacharya D. Adenosine deamination in human transcripts generates novel microRNA binding sites. Hum Mol Genet. (2009) 18:4801–7. doi: 10.1093/hmg/ddp443, PMID: 19776031 PMC2778373

[16] Fei J Cui XB Wang JN Dong K Chen SY. ADAR1-mediated RNA editing, a novel mechanism controlling phenotypic modulation of vascular smooth muscle cells. Circ Res. (2016) 119:463–9. doi: 10.1161/circresaha.116.309003, PMID: 27199464 PMC4961590

[17] Grefte S Kuijpers-Jagtman AM Torensma R von den Hoff JW. Skeletal muscle development and regeneration. Stem Cells Dev. (2007) 16:857–68. doi: 10.1089/scd.2007.0058 17999606

[18] Bismuth K Relaix F. Genetic regulation of skeletal muscle development. Exp Cell Res. (2010) 316:3081–6. doi: 10.1016/j.yexcr.2010.08.018 20828559

[19] Millay DP. Regulation of the myoblast fusion reaction for muscle development, regeneration, and adaptations. Exp Cell Res. (2022) 415:113134. doi: 10.1016/j.yexcr.2022.113134, PMID: 35367215 PMC9058940

[20] Fukada SI Higashimoto T Kaneshige A. Differences in muscle satellite cell dynamics during muscle hypertrophy and regeneration. Skelet Muscle. (2022) 12:17. doi: 10.1186/s13395-022-00300-0, PMID: 35794679 PMC9258228

[21] Buckingham M Rigby PW. Gene regulatory networks and transcriptional mechanisms that control myogenesis. Dev Cell. (2014) 28:225–38. doi: 10.1016/j.devcel.2013.12.020 24525185

[22] Comai G Tajbakhsh S. Molecular and cellular regulation of skeletal myogenesis. Curr Top Dev Biol. (2014) 110:1–73. doi: 10.1016/b978-0-12-405943-6.00001-4 25248473

[23] Yu X Wang Z Sun H Yang Y Li K Tang Z. Long non-coding MEG3 is a marker for skeletal muscle development and meat production traits in pigs. Anim Genet. (2018) 49:571–8. doi: 10.1111/age.12712, PMID: 30294799

[24] Zhang J Ying ZZ Tang ZL Long LQ Li K. MicroRNA-148a promotes myogenic differentiation by targeting the ROCK1 gene. J Biol Chem. (2012) 287:21093–101. doi: 10.1074/jbc.M111.330381, PMID: 22547064 PMC3375532

[25] Yan J Yang Y Fan X Liang G Wang Z Li J. circRNAome profiling reveals circFgfr2 regulates myogenesis and muscle regeneration via a feedback loop. J Cachexia Sarcopenia Muscle. (2022) 13:696–712. doi: 10.1002/jcsm.12859, PMID: 34811940 PMC8818660

[26] McKinnell IW Ishibashi J Le Grand F Punch VG Addicks GC Greenblatt JF. Pax7 activates myogenic genes by recruitment of a histone methyltransferase complex. Nat Cell Biol. (2008) 10:77–84. doi: 10.1038/ncb1671, PMID: 18066051 PMC2739814

[27] Yang Y Fan X Yan J Chen M Zhu M Tang Y. A comprehensive epigenome atlas reveals DNA methylation regulating skeletal muscle development. Nucleic Acids Res. (2021) 49:1313–29. doi: 10.1093/nar/gkaa1203, PMID: 33434283 PMC7897484

[28] Chen J Liu HF Qiao LB Wang FB Wang L Lin Y. Global RNA editing identification and characterization during human pluripotent-to-cardiomyocyte differentiation. Mol Ther Nucleic Acids. (2021) 26:879–91. doi: 10.1016/j.omtn.2021.10.001, PMID: 34760335 PMC8551472

[29] Yang Y Zhu M Fan X Yao Y Yan J Tang Y. Developmental atlas of the RNA editome in *Sus scrofa* skeletal muscle. DNA Res. (2019) 26:261–72. doi: 10.1093/dnares/dsz006, PMID: 31231762 PMC6589548

[30] Picardi E Manzari C Mastropasqua F Aiello I D’Erchia AM Pesole G. Profiling RNA editing in human tissues: towards the inosinome atlas. Sci Rep. (2015) 5:14941. doi: 10.1038/srep14941, PMID: 26449202 PMC4598827

[31] Zhan S Zhai H Tang M Xue Y Li D Wang L. Profiling and functional analysis of mRNAs during skeletal muscle differentiation in goats. Animals. (2022) 12:1048. doi: 10.3390/ani12081048, PMID: 35454294 PMC9024908

[32] Quinlan AR Hall IM. BEDTools: a flexible suite of utilities for comparing genomic features. Bioinformatics. (2010) 26:841–2. doi: 10.1093/bioinformatics/btq033, PMID: 20110278 PMC2832824

[33] Eggington JM Greene T Bass BL. Predicting sites of ADAR editing in double-stranded RNA. Nat Commun. (2011) 2:319. doi: 10.1038/ncomms1324, PMID: 21587236 PMC3113232

[34] Magnaye KM Naughton KA Huffman J Hogarth DK Naureckas ET White SR. A-to-I editing of miR-200b-3p in airway cells is associated with moderate-to-severe asthma. Eur Respir J. (2021) 58:2003862. doi: 10.1183/13993003.03862-2020, PMID: 33446603

[35] Nakano M Fukami T Gotoh S Takamiya M Aoki Y Nakajima M. RNA editing modulates human hepatic aryl hydrocarbon receptor expression by creating microRNA recognition sequence. J Biol Chem. (2016) 291:894–903. doi: 10.1074/jbc.M115.699363, PMID: 26601943 PMC4705407

[36] Paul PK Gupta SK Bhatnagar S Panguluri SK Darnay BG Choi Y. Targeted ablation of TRAF6 inhibits skeletal muscle wasting in mice. J Cell Biol. (2010) 191:1395–411. doi: 10.1083/jcb.201006098, PMID: 21187332 PMC3010064

[37] Qiu J Zhu J Zhang R Liang W Ma W Zhang Q. miR-125b-5p targeting TRAF6 relieves skeletal muscle atrophy induced by fasting or denervation. Ann Transl Med. (2019) 7:456. doi: 10.21037/atm.2019.08.39, PMID: 31700892 PMC6803201

[38] Shtrichman R Germanguz I Mandel R Ziskind A Nahor I Safran M. Altered A-to-I RNA editing in human embryogenesis. PLoS One. (2012) 7:e41576. doi: 10.1371/journal.pone.0041576, PMID: 22859999 PMC3409221

[39] Wu X Chu M Ma X Pei J Xiong L Guo X. Genome-wide identification of RNA editing sites affecting muscle development in yak. Front Vet Sci. (2022) 9:871814. doi: 10.3389/fvets.2022.871814 35836505 PMC9274240

[40] Dumont NA Bentzinger CF Sincennes MC Rudnicki MA. Satellite cells and skeletal muscle regeneration. Compr Physiol. (2015) 5:1027–59. doi: 10.1002/cphy.c140068 26140708

[41] Peng Z Cheng Y Tan BC Kang L Tian Z Zhu Y. Comprehensive analysis of RNA-Seq data reveals extensive RNA editing in a human transcriptome. Nat Biotechnol. (2012) 30:253–60. doi: 10.1038/nbt.2122, PMID: 22327324

[42] Ramaswami G Li JB. RADAR: a rigorously annotated database of A-to-I RNA editing. Nucleic Acids Res. (2014) 42:D109–13. doi: 10.1093/nar/gkt996, PMID: 24163250 PMC3965033

[43] Li L Xu X Xiao M Huang C Cao J Zhan S. The profiles and functions of RNA editing sites associated with high-altitude adaptation in goats. Int J Mol Sci. (2023) 24:3115. doi: 10.3390/ijms24043115, PMID: 36834526 PMC9964554

[44] Shafiei H Bakhtiarizadeh MR Salehi A. Large-scale potential RNA editing profiling in different adult chicken tissues. Anim Genet. (2019) 50:460–74. doi: 10.1111/age.12818, PMID: 31355950

[45] Ramaswami G Zhang R Piskol R Keegan LP Deng P O’Connell MA. Identifying RNA editing sites using RNA sequencing data alone. Nat Methods. (2013) 10:128–32. doi: 10.1038/nmeth.2330, PMID: 23291724 PMC3676881

[46] Noda Y Okada S Suzuki T. Regulation of A-to-I RNA editing and stop codon recoding to control selenoprotein expression during skeletal myogenesis. Nat Commun. (2022) 13:2503. doi: 10.1038/s41467-022-30181-2, PMID: 35523818 PMC9076623

[47] Peng H Liu S Li Y Wang C Zhong Y. A novel circUBR4/miR-491-5p/NRP2 ceRNA network regulates oxidized low-density lipoprotein-induced proliferation and migration in vascular smooth muscle cells. J Cardiovasc Pharmacol. (2022) 79:512–22. doi: 10.1097/fjc.0000000000001204, PMID: 34935701

[48] Kyei B Odame E Li L Yang L Zhan S Li J. Knockdown of CDR1as decreases differentiation of goat skeletal muscle satellite cells via upregulating miR-27a-3p to inhibit ANGPT1. Genes. (2022) 13:663. doi: 10.3390/genes13040663, PMID: 35456469 PMC9026999

[49] Hudson MB Rahnert JA Zheng B Woodworth-Hobbs ME Franch HA Price SR. miR-182 attenuates atrophy-related gene expression by targeting FoxO3 in skeletal muscle. Am J Physiol Cell Physiol. (2014) 307:C314–9. doi: 10.1152/ajpcell.00395.2013, PMID: 24871856 PMC4137139

[50] Gao M Li X Yang Z Zhao S Ling X Li J. circHIPK3 regulates proliferation and differentiation of myoblast through the miR-7/TCF12 pathway. J Cell Physiol. (2021) 236:6793–805. doi: 10.1002/jcp.30363, PMID: 33748999

[51] Zhang G Chen F Wu P Li T He M Yin X. MicroRNA-7 targets the KLF4 gene to regulate the proliferation and differentiation of chicken primary myoblasts. Front Genet. (2020) 11:842. doi: 10.3389/fgene.2020.00842, PMID: 33193566 PMC7530283

[52] White AT Schenk S. NAD^+^/NADH and skeletal muscle mitochondrial adaptations to exercise. Am J Physiol Endocrinol Metab. (2012) 303:E308–21. doi: 10.1152/ajpendo.00054.2012, PMID: 22436696 PMC3423123

[53] Chal J Pourquié O. Making muscle: skeletal myogenesis *in vivo* and *in vitro*. Development. (2017) 144:2104–22. doi: 10.1242/dev.151035, PMID: 28634270

[54] Umansky KB Gruenbaum-Cohen Y Tsoory M Feldmesser E Goldenberg D Brenner O. Runx1 transcription factor is required for myoblasts proliferation during muscle regeneration. PLoS Genet. (2015) 11:e1005457. doi: 10.1371/journal.pgen.1005457, PMID: 26275053 PMC4537234

[55] Xie S Liu Q Fu C Chen Y Li M Tian C. Molecular regulation of porcine skeletal muscle development: insights from research on CDC23 expression and function. Int J Mol Sci. (2024) 25:3664. doi: 10.3390/ijms25073664, PMID: 38612477 PMC11011816

[56] Ono Y Sensui H Okutsu S Nagatomi R. Notch2 negatively regulates myofibroblastic differentiation of myoblasts. J Cell Physiol. (2007) 210:358–69. doi: 10.1002/jcp.20838, PMID: 17044085

[57] Jin W Liu M Peng J Jiang S. Function analysis of Mef2c promoter in muscle differentiation. Biotechnol Appl Biochem. (2017) 64:647–56. doi: 10.1002/bab.1524 27354201

[58] Pansters NA Schols AM Verhees KJ de Theije CC Snepvangers FJ Kelders MC. Muscle-specific GSK-3β ablation accelerates regeneration of disuse-atrophied skeletal muscle. Biochim Biophys Acta. (2015) 1852:490–506. doi: 10.1016/j.bbadis.2014.12.006, PMID: 25496993

[59] Verhees KJ Pansters NA Schols AM Langen RC. Regulation of skeletal muscle plasticity by glycogen synthase kinase-3β: a potential target for the treatment of muscle wasting. Curr Pharm Des. (2013) 19:3276–98. doi: 10.2174/1381612811319180011 23151136

[60] Kim D Hwang HY Ji ES Kim JY Yoo JS Kwon HJ. Activation of mitochondrial TUFM ameliorates metabolic dysregulation through coordinating autophagy induction. Commun Biol. (2021) 4:1. doi: 10.1038/s42003-020-01566-0, PMID: 33398033 PMC7782552

[61] Qiu S Li W Xiong H Liu D Bai Y Wu K. Single-cell RNA sequencing reveals dynamic changes in A-to-I RNA editome during early human embryogenesis. BMC Genomics. (2016) 17:766. doi: 10.1186/s12864-016-3115-2, PMID: 27687780 PMC5043600

[62] Watt KI Goodman CA Hornberger TA Gregorevic P. The hippo signaling pathway in the regulation of skeletal muscle mass and function. Exerc Sport Sci Rev. (2018) 46:92–6. doi: 10.1249/jes.0000000000000142, PMID: 29346163 PMC6319272

[63] Norris AM Appu AB Johnson CD Zhou LY McKellar DW Renault MA. Hedgehog signaling via its ligand DHH acts as cell fate determinant during skeletal muscle regeneration. Nat Commun. (2023) 14:3766. doi: 10.1038/s41467-023-39506-1, PMID: 37355632 PMC10290686

[64] Kurisaki T Masuda A Osumi N Nabeshima Y Fujisawa-Sehara A. Spatially-and temporally-restricted expression of meltrin alpha (ADAM12) and beta (ADAM19) in mouse embryo. Mech Dev. (1998) 73:211–5. doi: 10.1016/s0925-4773(98)00043-4, PMID: 9622634

[65] Kurisaki T Masuda A Sudo K Sakagami J Higashiyama S Matsuda Y. Phenotypic analysis of meltrin alpha (ADAM12)-deficient mice: involvement of meltrin alpha in adipogenesis and myogenesis. Mol Cell Biol. (2003) 23:55–61. doi: 10.1128/mcb.23.1.55-61.2003, PMID: 12482960 PMC140658

[66] Galliano MF Huet C Frygelius J Polgren A Wewer UM Engvall E. Binding of ADAM12, a marker of skeletal muscle regeneration, to the muscle-specific actin-binding protein, alpha -actinin-2, is required for myoblast fusion. J Biol Chem. (2000) 275:13933–9. doi: 10.1074/jbc.275.18.13933, PMID: 10788519

[67] Stupka N Kintakas C White JD Fraser FW Hanciu M Aramaki-Hattori N. Versican processing by a disintegrin-like and metalloproteinase domain with thrombospondin-1 repeats proteinases-5 and -15 facilitates myoblast fusion. J Biol Chem. (2013) 288:1907–17. doi: 10.1074/jbc.M112.429647, PMID: 23233679 PMC3548499

[68] Yang X Ning Y Abbas Raza SH Mei C Zan L. MEF2C expression is regulated by the post-transcriptional activation of the METTL3-m^6^A-YTHDF1 axis in myoblast differentiation. Front Vet Sci. (2022) 9:900924. doi: 10.3389/fvets.2022.900924, PMID: 35573410 PMC9096896

