# Supplementary material for: Global A-to-I RNA editing during myogenic differentiation of goat MuSCs
Source: Front Vet Sci. 2024 Oct 9;11:1439029. doi: 10.3389/fvets.2024.1439029 (PMC11496035; doi:10.3389/fvets.2024.1439029)
Supplement: Supplementary file 1 [file Supplementary_Data_Sheet_1.DOCX]

Supplementary Material


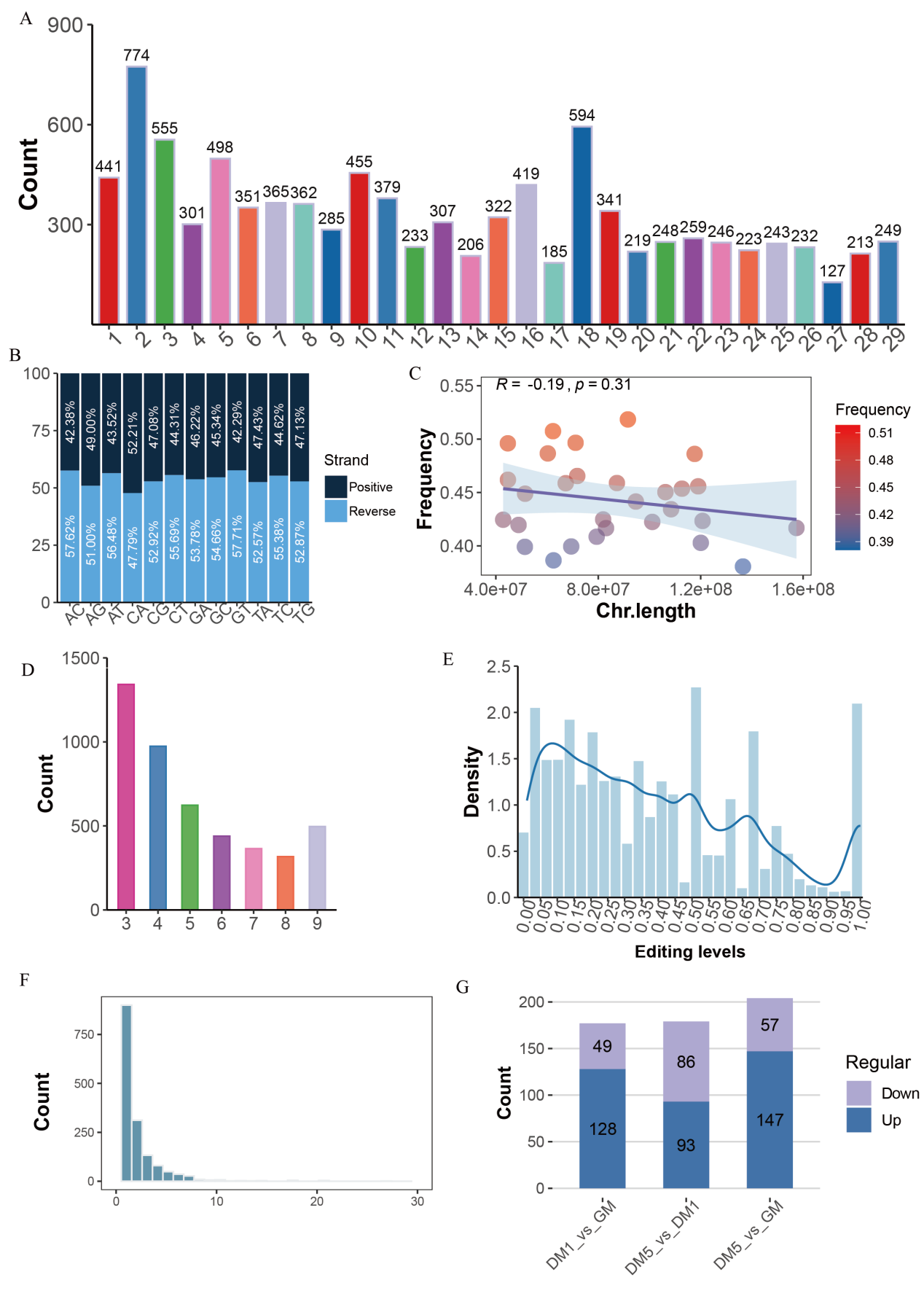


**Figure S1** A. The editing numbers of RNA editing sites across chromosomes. B. The proportion of 12 editing types between the positive strand and reverse strand. C. The relationship between chromosome length and the levels of editing sites. D. The count of A-to-I editing sites across different intervals of samples. E. The distribution of A-to-I editing levels. F. The numbers of RNA editing sites occurred per gene. G. The number of down-regulated and up-regulated differential A-to-I editing sites.

**
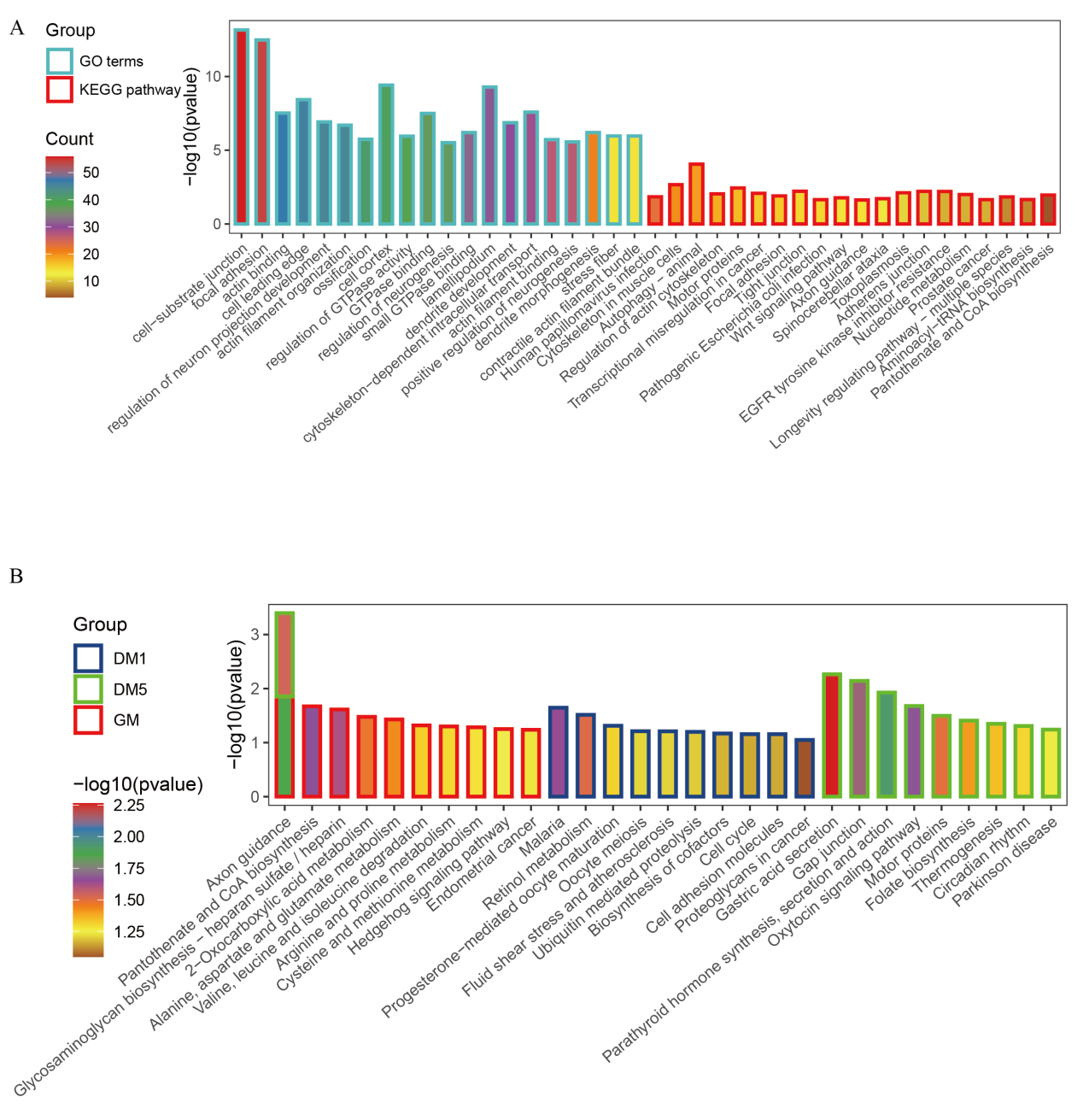
**

**Figure S2** A. GO terms and KEGG pathways of genes with shared editing sites in each stage. B. KEGG pathways of genes with stage-specific A-to-I editing sites.


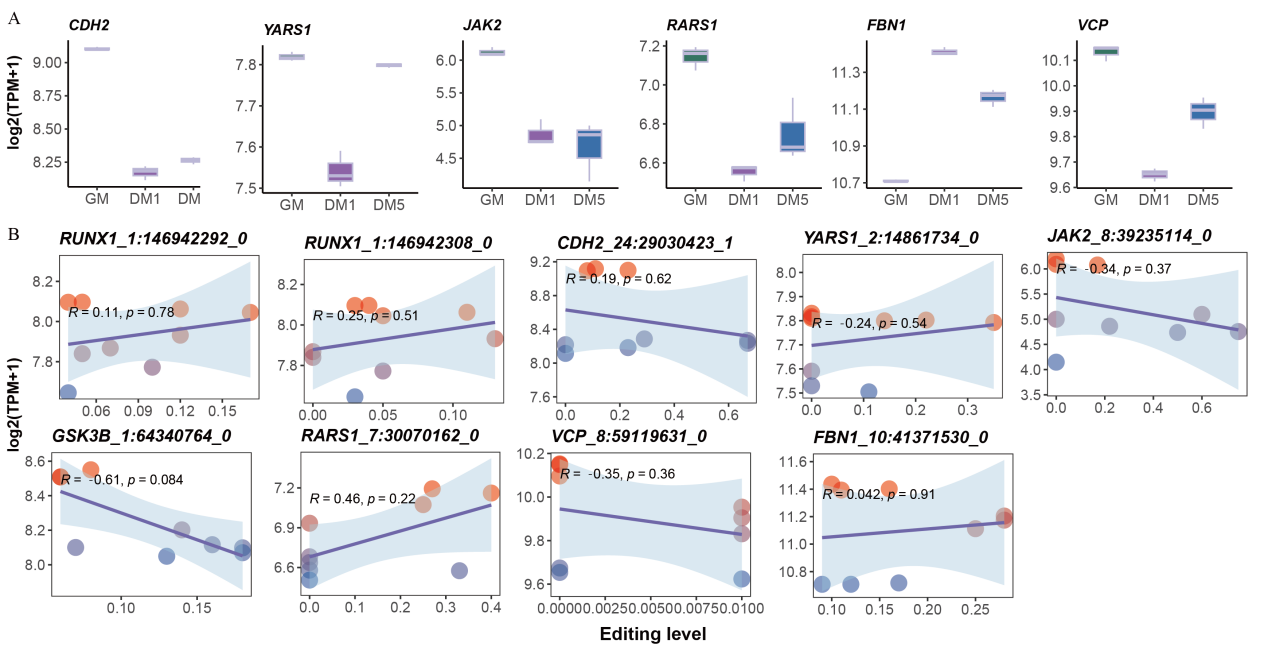


**Figure S3** A. The expression of 6 hub genes with differential A-to-I editing sites in each sample. B. The interaction relationship between expression levels of 8 hub genes and editing levels of differential A-to-I editing sites.

**Table S**

Table S1. List of high-quality RNA editing sites across 9 individuals.

Table S2. List of A-to-I RNA editing sites.

Table S3. GO enrichment of protein-coding genes involved in A-to-I RNA editing sites.

Table S4. KEGG enrichment of protein-coding genes involved in A-to-I RNA editing sites.

Table S5. List of A-to-I editing sites function annotation.

Table S6. Effects of RNA editing on miRNA-binding sites in the 3’UTR of genes.

Table S7. Differential A-to-I editing sites.

Table S8. GO enrichment of differential and specific A-to-I editing sites.

Table S9. Specific A-to-I editing sites.
